# Divergent Molecular Responses to Heavy Water in *Arabidopsis thaliana* Compared to Bacteria and Yeast

**DOI:** 10.3390/plants13223121

**Published:** 2024-11-06

**Authors:** Pengxi Wang, Jan Novák, Romana Kopecká, Petr Čičmanec, Martin Černý

**Affiliations:** Department of Molecular Biology and Radiobiology, Faculty of AgriSciences, Mendel University in Brno, 61300 Brno, Czech Republic; penxiwang@gmail.com (P.W.); novakhonza@atlas.cz (J.N.); petr.cicmanec@seznam.cz (P.Č.)

**Keywords:** stress response, proteome, ROS metabolism, deuterium oxide, adaptation, HSP70

## Abstract

Heavy water (D_2_O) is scarce in nature, and despite its physical similarity to water, D_2_O disrupts cellular function due to the isotope effect. While microbes can survive in nearly pure D_2_O, eukaryotes such as *Arabidopsis thaliana* are more sensitive and are unable to survive higher concentrations of D_2_O. To explore the underlying molecular mechanisms for these differences, we conducted a comparative proteomic analysis of *E. coli*, *S. cerevisiae*, and *Arabidopsis* after 180 min of growth in a D_2_O-supplemented media. Shared adaptive mechanisms across these species were identified, including changes in ribosomal protein abundances, accumulation of chaperones, and altered metabolism of polyamines and amino acids. However, *Arabidopsis* exhibited unique vulnerabilities, such as a muted stress response, lack of rapid activation of reactive oxygen species metabolism, and depletion of stress phytohormone abscisic acid signaling components. Experiments with mutants show that modulating the HSP70 pool composition may promote D_2_O resilience. Additionally, *Arabidopsis* rapidly incorporated deuterium into sucrose, indicating that photosynthesis facilitates deuterium intake. These findings provide valuable insights into the molecular mechanisms that dictate differential tolerance to D_2_O across species and lay the groundwork for further studies on the biological effects of uncommon isotopes, with potential implications for biotechnology and environmental science.

## 1. Introduction

Heavy water (D_2_O) represents only a minute fraction of the water naturally found on Earth. With the natural abundance of deuterium (D) being approximately 0.015% [1], the likelihood of encountering a fully deuterated water molecule is about 1 in 44 million. If hydrogen exchange were to be disregarded and the D_2_O content within a cell estimated purely based on water volume and the calculated likelihood of occurrence, a bacterium the size of *E. coli* (≈1 µm^3^) with approximately 70% water content would contain roughly 500 molecules of D_2_O. It is physically and chemically almost identical to ordinary water, with a 10% increase in density of its liquid form and a small increase in the freezing and boiling points (https://pubchem.ncbi.nlm.nih.gov/compound/24602; accessed on 15 August 2024). However, it is not functionally equivalent to biochemical reactions, as a hydrogen bond in D_2_O is stronger, and the covalent bond between oxygen and deuterium shorter, while the intermolecular hydrogen bond is longer [2].

In effect, the substitution of H_2_O with D_2_O induces significant alterations in reaction kinetics and macromolecular structures, a phenomenon known as the solvent isotope effect. It also affects solubility of metabolites like carbohydrates and amino acids [3,4]. Additionally, the metabolic incorporation of deuterium into biomolecules, along with spontaneous protium–deuterium exchange, exerts a profound influence on macromolecular properties, termed the deuterium isotope effect [5]. These isotopic effects can disrupt normal cellular functions, leading to inhibited growth and, at higher concentrations, lethality in living organisms. The biological impact of heavy water on living organisms is largely hypothetical. In practice, significant exposure to D_2_O is unlikely outside of industrial accidents or nuclear power plant leaks, and the concentrations required to exert harmful effects are not typically encountered in natural or laboratory environments. However, it serves as an intriguing tool for investigating metabolism and for obtaining labeled compounds, and it has been utilized in bioassays ever since its discovery.

Microorganisms exhibit varying degrees of resistance to D_2_O. Notably, *Escherichia coli* and *Saccharomyces cerevisiae* demonstrate remarkable adaptability, surviving in environments with 100% and 90% D_2_O, respectively [6,7]. However, the cultivation of *E. coli* in 70% D_2_O leads to a substantial increase in generation time, extending it by more than 1.4-fold [8]. A similar retardation effect is observed in *Schizosaccharomyces pombe* [9]. Interestingly, this growth delay may confer a longevity benefit by reducing the endogenous production of reactive oxygen species [10]. Multicellular organisms are notably more susceptible to the effects of heavy water. In plants, D_2_O concentrations exceeding 30% inhibit critical developmental processes, including germination, root growth, and hypocotyl elongation. Furthermore, D_2_O exposure modulates the circadian clock and induces general growth retardation [11,12,13,14]. The model plant *Arabidopsis thaliana* fails to survive on media containing 50% D_2_O; however, subsequent generations display adaptive capacity, with growth observed on media containing up to 70% D_2_O [15]. Despite its low natural abundance, D_2_O has long attracted scientific attention. It serves as a valuable tool for metabolite labeling, both for tracing metabolic pathways and obtaining labeled standards [14,16]. Additionally, D_2_O provides unique insights into molecular mechanisms [2], and some results could be quite surprising, as documented in a recent study that identified G-protein receptors in human cells that specifically interact with D2O, explaining its reportedly sweeter taste [17]. However, the molecular mechanisms underlying its toxicity in certain species remain largely unexplored.

In this study, we present a comprehensive analysis of *A. thaliana*’s response to D_2_O, leveraging the best-characterized model plant organism. We investigate the early response to heavy water in three model organisms: D_2_O-resistant bacteria (*E. coli*), yeast (*S. cerevisiae*), and the model plant *A. thaliana*. We assess the incorporation of deuterium into selected metabolites, highlight D_2_O-responsive proteins, and investigate potential mechanisms to explain why *Arabidopsis* struggles to adapt to D_2_O exposure. To the best of our knowledge, this is the first attempt to compare the early responses of these diverse model organisms to heavy water treatment.

## 2. Results

### 2.1. Rapid Incorporation of Deuterium into Primary Metabolites Across Model Organisms

**Figure 1:**
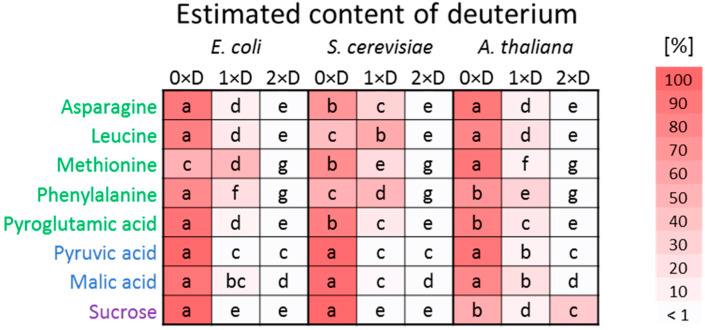
Deuterium incorporation into primary metabolites following three hours of D_2_O exposure. The heatmap depicts mean relative abundances of the three most abundant isotopologue states (XH_n_, XH_n−1_D_1_, and XH_n−2_D_2_) for each metabolite. Data represent means of at least three biological replicates. Different letters indicate significant differences between isotopologue states (Kruskal–Wallis and Conover’s test, *p* < 0.05). See Supplementary Tables S1 and S2 for details.

To investigate the metabolic effects of heavy water (D_2_O) on model organisms, we conducted gas chromatography–mass spectrometry (GC–MS) analysis of polar metabolites isolated from three model organisms, namely, yeast (*Saccharomyces cerevisiae*), bacteria (*Escherichia coli*), and plant (*Arabidopsis thaliana*) incubated for three hours in nutrition media prepared in D_2_O. We focused on eight key metabolites with known rapid turnover rates, selecting them for detailed isotopologue profiling. These metabolites, encompassing amino acids, tricarboxylic acid cycle intermediates, and sucrose, were examined across three organisms (Figure 1). Our results demonstrate that a three-hour D_2_O treatment was sufficient to incorporate deuterium into metabolites across all three species. However, the extent of deuterium incorporation exhibited species-specific variations. For instance, leucine and phenylalanine showed the highest deuterium incorporation in *S. cerevisiae*, consistent with the organism’s higher turnover rates compared to *E. coli* [18]. Interestingly, methionine isotopologues containing deuterium were most abundant in *E. coli*, with moderate incorporation in *S. cerevisiae* (24.3 ± 3.8%) and minimal in Arabidopsis (8.2 ± 2.4%). Malic acid showed minimal deuterium incorporation, while deuterated pyruvate met the threshold (≥1% of total estimated abundance) only in *Arabidopsis*. Notably, sucrose was the sole metabolite with substantial isotopologue formation containing two deuterium atoms, observed exclusively in *Arabidopsis*. In summary, this experiment confirms the impact of D_2_O on the organisms under the given experimental conditions, highlighting differences that can be attributed to contrasting metabolic strategies.

### 2.2. Proteomic Analysis Reveals D_2_O-Induced Stress Responses and Inhibited Growth in E. coli

A total of 1881 and 1438 *E. coli* proteins were identified and quantified, respectively. A comparison between D_2_O- and H_2_O-treated bacteria revealed 362 differentially abundant proteins (DAPs; adjusted *p*-value < 0.05, absolute fold change > 1.4; Supplementary Table S3), representing 27.5% of the estimated protein abundance in control bacteria. Gene ontology enrichment analysis indicated that the most significant impact was on protein translation, including ribosome biogenesis and the regulation of transcription and translation, encompassing more than 30 DAPs, all of which showed a significant decrease in abundance in D_2_O-treated bacteria. The subset of proteins that accumulated in response to D_2_O included multiple components of the oxidative stress response, such as periplasmic superoxide dismutase SodC, acetaldehyde dehydrogenase AdhE (which acts as a hydrogen peroxide scavenger under aerobic conditions), peptide methionine sulfoxide reductase MsrB (involved in the repair of oxidatively damaged proteins), the redox-regulated chaperone Hsp33, and protein/nucleic acid deglycase (which prevents the formation of advanced glycation end-products [19]), and WrbA (which reduces quinones to the hydroquinone state to prevent the production of superoxide under stress). The analysis of documented mutant phenotypes corroborated the expected impact of D_2_O on growth. DAPs that were significantly less abundant in treated bacteria included proteins essential for viability, such as GTPase Era [20], ribosomal silencing factor RsfS [21], and tRNA threonylcarbamoyladenosine biosynthesis protein TsaB [22]. Proteins required for maintaining growth, such as RNA helicase DeaD, polyribonucleotide nucleotidyltransferase [23], lipopolysaccharide export system protein LptA [24], and proteins critical for growth under suboptimal conditions, such as 30S ribosome-binding factor [25] and inositol-1-monophosphatase [26], were also less abundant.

### 2.3. Heavy-Water-Induced Stress Response in S. cerevisiae

A total of 2237 and 1592 *S. cerevisiae* proteins were identified and quantified, respectively. A comparison between D_2_O- and H_2_O-treated yeast revealed 207 DAPs (Supplementary Table S4), representing 3.6% of the estimated protein abundance in the control yeast culture. Analysis of the identified DAPs indicated that the response to heavy water involved alterations in energy metabolism, amino acid metabolism, proteosynthesis, ribosome biogenesis, cellular transport and trafficking, reactive oxygen species (ROS) metabolism, and stress response. Noteworthy proteins that showed a significant decrease in abundance included catalase T, superoxide dismutase [Mn], and glutathione-independent glyoxalase HSP31, indicating impaired protection against ROS [27]. Additional decreases were observed in the nucleosome assembly protein NAP1 and cyclin-dependent kinase 1, suggesting reduced mitotic activity, as well as in inositol-3-phosphate synthase, a key enzyme in myo-inositol biosynthesis (inositol is essential for cell viability [28]). The reduced abundance of altered inheritance of mitochondria protein 46 suggests impaired mitochondrial biogenesis [29], while Fe-S cluster assembly protein DRE2, essential for cell viability [30], and proteins involved in signal transduction, such as regulatory protein MIG1, were also significantly decreased.

### 2.4. Arabidopsis Proteome Showed Limited Response to D_2_O

A total of 3275 and 2176 *Arabidopsis thaliana* seedling proteins were identified and quantified, respectively. A comparison between D_2_O- and H_2_O-treated seedlings revealed 127 differentially abundant proteins (DAPs) (Supplementary Table S5), representing less than 2% of the estimated protein abundance in control plants. Despite the seemingly negligible impact of D_2_O on the seedling proteome, the identified DAPs clearly illustrated a response to stress and adjustments in metabolism. Detailed analysis indicated that D_2_O-responsive proteins are predominantly involved in photosynthesis, protein metabolism, stress response, sulfur metabolism, secondary metabolism, and transport (Supplementary Table S5). Proteins that increased in abundance included components of the photosynthesis machinery, such as chlorophyll a-b binding protein CP29.2, LHCB6, LHC-like protein AT4G17600, photosystem II reaction center proteins CP43 and 47, plastocyanin minor isoform, and PsbQ-like protein 2, along with proteins involved in protein import into chloroplasts, including TIC 62 and stromal processing peptidase. These findings suggest that heavy water may decrease the efficiency of photosynthesis. Additional indicators of stress response include changes in proteins involved in osmoprotectant metabolism, such as a decrease in the proline catabolism enzyme delta-1-pyrroline-5-carboxylate dehydrogenase 12A1, which correlates with the observed accumulation of proline (1.4-fold increase in abundance, *p* < 0.05; Supplementary Table S2), the accumulation of glutathione gamma-glutamylcysteinyltransferase 1 (mutant plants are highly sensitive to cadmium ions [31]), an increase in the abundance of heat shock protein 70-5, and variations in proteins associated with ROS metabolism, including dynamin-related protein 3B (impacts peroxisome size and number), glutathione S-transferases F10 and U1, monothiol glutaredoxin-S15, and succinate semialdehyde dehydrogenase AT1G79440. A decrease in nitrate reductase AT1G37130 was noted, which could coincide with reduced nitrate reductase activity to conserve energy and resources during the early response to stress. Heavy water also impacted abscisic acid signaling, as indicated by a decrease in its receptor PYL1, a negative regulator of abscisic acid responses ERD15 [32], and magnesium chelatase subunit ChlH [33].

### 2.5. Evolutionary Conserved Response to D_2_O Included Attenuated Proteosynthesis, Nucleotide Biosynthesis, and an Impact on Polyamine Metabolism

**Figure 2:**
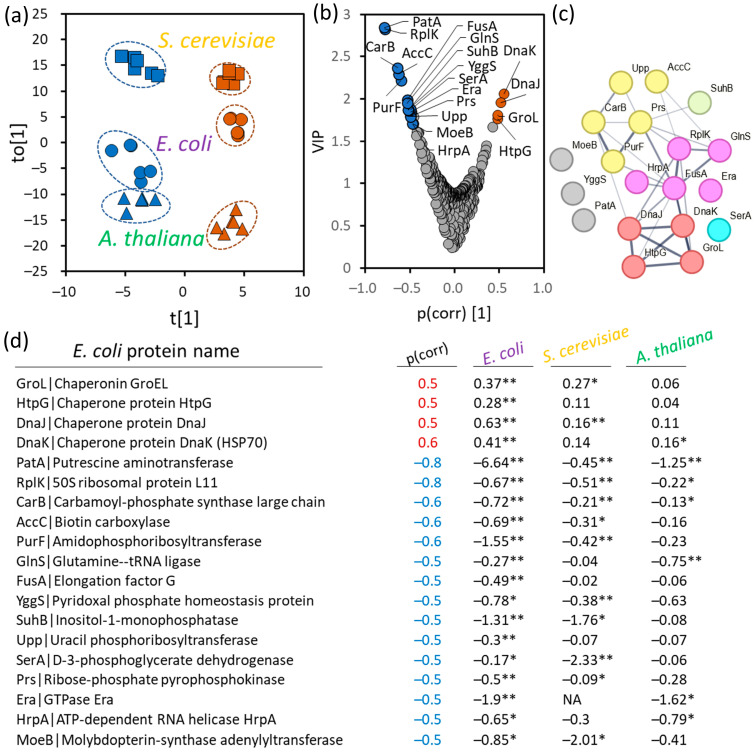
Evolutionarily conserved proteins found in response to heavy water treatment. (**a**,**b**) Orthogonal partial least squares (OPLS) model and the corresponding variable importance in projection (VIP) based on summed estimated protein abundances of 407 orthogonal proteins found in at least two model organisms treated with heavy water (brown) or standard water (blue); (**c**) functional enrichment interactions of identified proteins visualized by STRING [35]. The thickness of lines indicates the strength of data support; the minimum required interaction score is 0.4 (medium confidence). Red dots represent chaperons and protein folding; blue, amino acid biosynthesis; pink, protein metabolism; yellow, nucleotide biosynthesis; (**d**) identified proteins (based on *E. coli* orthologs) with absolute *p*(corr) > 0.5. Numbers indicate log_2_ fold-change values (*n* = 6) and the results of Student’s *t*-test (* <0.05, ** <0.01). For more details, see Supplementary Tables S3–S6.

To enable a direct comparison of the three datasets, proteins identified in *S. cerevisiae* and *A. thaliana* were cross-referenced with the *E. coli* proteome, and the abundances of putative isoforms were summed, yielding a set of 407 shared proteins (this analysis included all identified protein with at least detected unique peptide). The estimated abundances of these proteins were then analyzed using orthogonal partial least squares (OPLS) modeling (Figure 2a). Subsequent variable importance in projection (VIP; Figure 2b) analysis identified a subset of evolutionarily conserved proteins whose abundances correlated with D_2_O response. Notably, these conserved responses were predominantly associated with a negative regulation by D_2_O. The conserved response was characterized by attenuated proteosynthesis, evidenced by decreased levels of the 50S ribosomal protein (RplK), protein chain elongation factor (FusA), chaperone for 16S rRNA processing and 30S ribosomal subunit biogenesis Era, and glutamine tRNA synthetase GlnS (Figure 2b–d). Four proteins involved in nucleotide biosynthesis exhibited significant decreases in abundance within the bacterial and yeast proteomes (PurF, AccC, Prs, CarB), though only carbamoyl phosphate synthase (CarB) showed a statistically significant decrease in its *Arabidopsis* orthologs. Additionally, the data suggest an evolutionarily conserved response related to chaperons and protein folding. Orthologs of chaperons GroL, HtpG, DnaJ, and DnaK show an increase in abundance in response to heavy water, aligning well with the documented impact of heavy water on protein folding [2]. The response was again least manifested in *Arabidopsis*, but this was likely due to the summed abundances of all orthologs used for the projection, as HSP70-5 was significantly accumulated in D_2_O-treated seedlings (Supplementary Table S5). Another interesting pathway indicated by the analysis was polyamine metabolism. Putrescine aminotransferase (PutA), a key enzyme in polyamine metabolism, initiates putrescine degradation. Carbamoyl phosphate synthase (CarB), though indirectly linked, supports polyamine metabolism via ornithine production through the urea cycle, a precursor for polyamine synthesis. The role of polyamines was further underscored by observed changes in related proteins, including a decrease in yeast polyamine transporter 2 and bacterial polyamine export protein (Supplementary Tables S3 and S4), the latter’s mutation enhancing sensitivity to cadaverine and putrescine [34]. Metabolome profiling indicated a more than 1.5-fold increase in putrescine in *Arabidopsis* seedlings treated with D_2_O (Supplementary Table S2; *p* < 0.1), further supporting the significance of polyamine metabolism in D_2_O response.

### 2.6. Metabolic Pathways Impacted by D_2_O Show Only Limited Overlap Between Model Organisms

**Figure 3:**
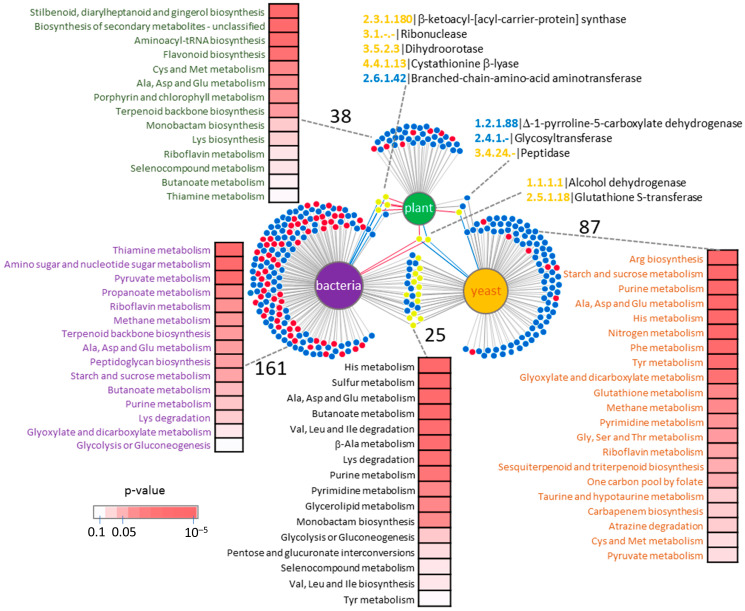
Heavy water impact on metabolism is predominantly organism-specific. The DiVenn visualization of identified differentially abundant enzymes specific to *E. coli* (bacteria), *S. cerevisiae* (yeast), and *A. thaliana* (plant). Red dots indicate a relative increase and decrease in protein abundances compared to corresponding controls in H_2_O, respectively, while yellow dots represent differential responses between the comparisons. Heat maps represent enriched metabolic pathways identified using MetaboAnalyst.

Orthologous protein searches may not fully capture the nuances of molecular mechanisms underlying the response to heavy water (D_2_O), as some isoforms lack direct orthology. To gain deeper insights, we compared the proteomic profiles and enzyme responses across our three datasets (Figure 3). Surprisingly, the overlap in differentially abundant enzymes was minimal. Only glutathione S-transferase and alcohol dehydrogenase were identified across all three datasets, yet their regulatory patterns varied significantly (Figure 3). Although there was limited overlap in individual enzymes, comparative analysis of enriched metabolic pathways identified additional shared features. Across all organisms studied, D_2_O consistently impacted riboflavin metabolism and the metabolism of amino acids Ala, Asp, and Glu. Bacteria and yeast exhibited shared regulation of pyruvate metabolism, methane metabolism, starch and sucrose metabolism, and purine metabolism, as well as glyoxylate and dicarboxylate metabolism. In contrast, thiamine metabolism, butanoate metabolism, and terpenoid biosynthesis were similarly affected in plants and bacteria, while the metabolism of sulfur-containing amino acids Cys and Met was conserved between plants and yeast. The pathways found to be significantly enriched exclusively in bacteria were primarily associated with core metabolic processes, including amino sugar/nucleotide sugar metabolism, and propanoate metabolism. Additionally, peptidoglycan biosynthesis, a hallmark of bacterial cell walls, was uniquely overrepresented in the *E. coli* proteome. Yeast exhibited a broader response, encompassing primary metabolic pathways such as amino acid, nitrogen, glutathione, and pyrimidine metabolism. Moreover, elements of secondary metabolism and xenobiotic degradation, and pathways involved in antibiotic biosynthesis were enriched. Conversely, pathways unique to *Arabidopsis* were of secondary metabolism with significant enrichment in stilbenoid, diarylheptanoid, gingerol, flavonoid biosynthesis, and porphyrin and chlorophyll metabolism (Figure 3).

### 2.7. Key Enzymes of ROS Metabolism Are Accumulated in Bacteria, Significantly Depleted in Yeast, and Not Affected in Plant Response to D_2_O

**Figure 4:**
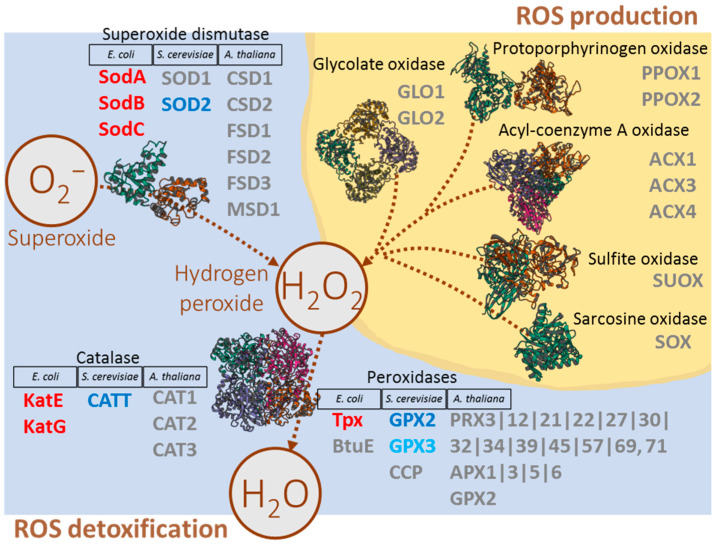
Reactive oxygen species metabolism. The figure illustrates the abundance changes in key ROS-metabolizing enzymes in *E. coli*, *S. cerevisiae*, and *A. thaliana* in response to heavy water exposure. Red indicates an increase in enzyme abundance, blue denotes a decrease, and grey represents no significant change. Protein structures were obtained from https://www.rcsb.org/ (accessed on 15 August 2024). For more detailed information, refer to Supplementary Table S7.

Many published studies indicate that ROS plays a role in the response to heavy water. Therefore, we filtered out ROS metabolism enzymes from our dataset and evaluated their response to D_2_O treatment (Figure 4, Supplementary Table S7). All three quantified superoxide dismutases were significantly more abundant in D_2_O-treated E. coli, and an accumulation was also observed for catalase and two peroxidases. In contrast, mitochondrial superoxide dismutase in yeast, along with catalase and glutathione peroxidase, were significantly less abundant in response to D_2_O. This aligns well with the potential impaired mitochondria biogenesis indicated by the observed DAPs and is consistent with previously published findings showing a significant decrease in ROS production in yeast grown on D_2_O-containing medium [10]. The proteomic analysis of *Arabidopsis* revealed 40 ROS metabolism enzymes [36], but differences were only observed in glutathione S-transferases, with no apparent effect on other ROS metabolic enzymes.

### 2.8. Mutation in HSP70 Attenuates D_2_O-Induced Root Growth Inhibition in Arabidopsis

**Figure 5:**
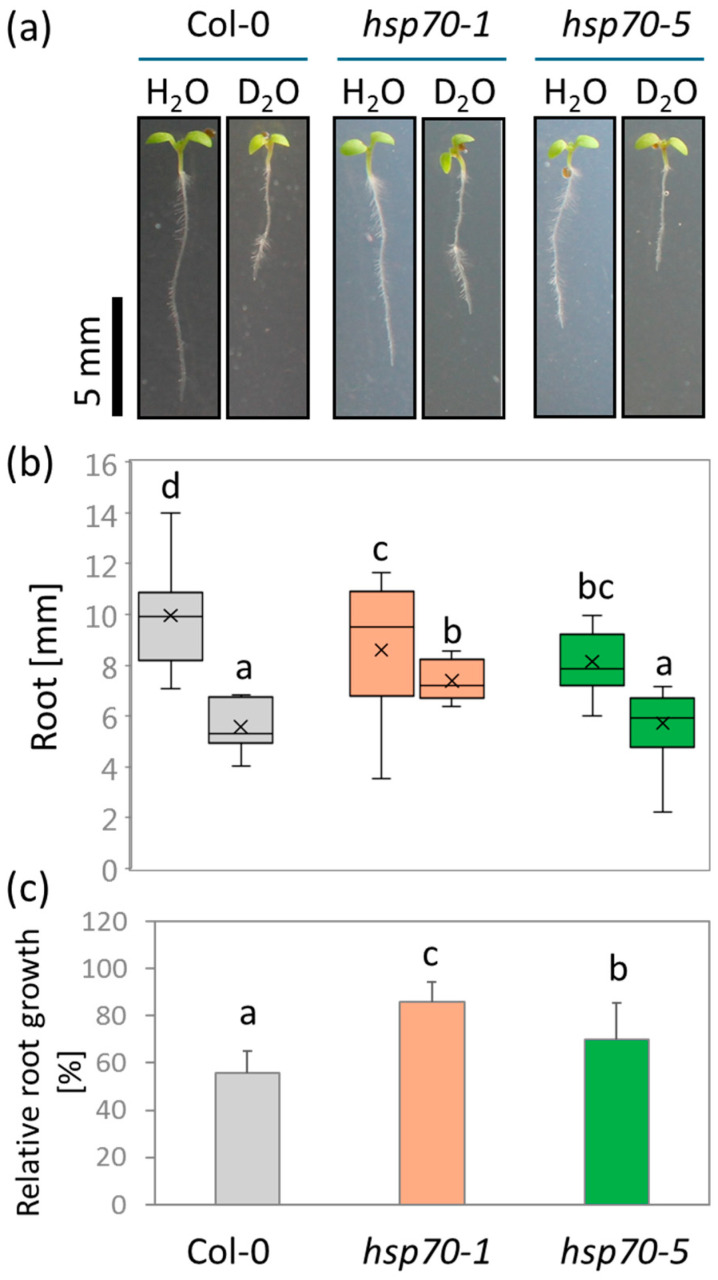
D_2_O-induced root inhibition is reduced in *hsp70* mutant lines. (**a**) Representative images of seven-day-old seedlings grown on standard medium (H_2_O) and medium supplemented with 30% D_2_O. (**b**) Absolute root length visualized in a box plot. The line and cross within the box represent the median and mean, respectively; (**c**) relative root length (normalized to respective controls) shown as means ± standard deviations. Significant differences are indicated by different letters (Kruskal–Wallis and Conover’s test, *p* < 0.05; based on two biological replicates, each with *n* = 10 seedlings).

The impact of D_2_O on chaperones and its relative absence in *Arabidopsis* prompted us to analyze this in greater detail, with a focus on the HSP70 protein family. A total of 13 HSP70 isoforms were quantified in *Arabidopsis seedlings* (Supplementary Table S7). Among these, three isoforms (5, 9, and 11) showed a statistically significant increase in abundance (*p* < 0.05), but only HSP70-5 met the set fold change threshold. Together, these three isoforms accounted for less than 8% of the estimated protein abundance of the HSP70 protein family in control seedlings. To investigate the role of HSP70 in response to D_2_O, we analyzed root elongation in mutants of *HSP70-5* and the dominant isoform *HSP70-1*, which accounted for nearly 30% of the estimated protein abundance of the HSP70 protein family and, like HSP70-5, is also located in the cytosol. Given that the HSP70-mediated response cannot fully account for all the observed differences, we modified the experimental design to assess its impact on growth parameters during prolonged exposure to a non-lethal dose of D_2_O. Mutations in *HSP70* adversely affected root growth, with *hsp70-5* and *hsp70-1* seedlings achieving only 82% and 86% of the root length of Col-0, respectively (Figure 5a,b). Exposure to 30% D_2_O significantly inhibited root growth, though the effect of D_2_O was notably less severe in *hsp70-1*. When comparing relative differences, the impact of D_2_O was most pronounced in Col-0, with root length reduced to just 45% of the control. In contrast, the mutants retained 55% and 66% of their corresponding control root lengths for *hsp70-5* and *hsp70-1*, respectively (Figure 5c).

### 2.9. Mutation in HSP70 May Promote ROS Metabolism and Alleviate D_2_O-Induced Inhibition in Arabidopsis

**Figure 6:**
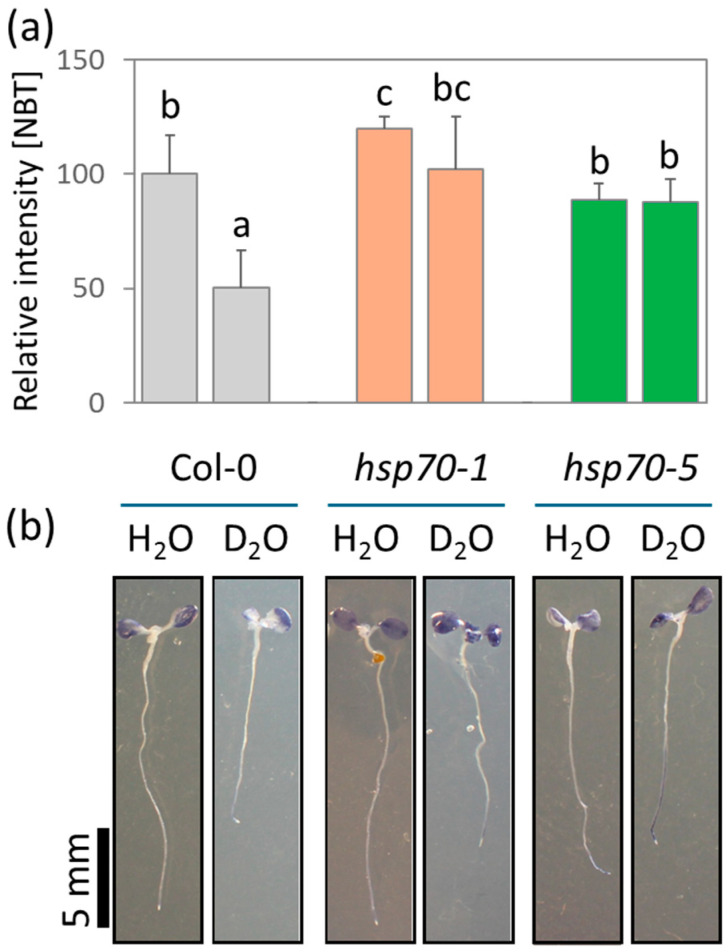
Heavy water impact on superoxide radical production as estimated by histochemical staining with nitroblue tetrazolium (NBT). (**a**) Relative intensity of NBT stain (normalized to Col-0 controls) shown as means ± standard deviations. Significant differences are indicated by different letters (Kruskal–Wallis and Conover’s test, *p* < 0.05; *n* = 5); (**b**) representative images of ten-day-old seedlings grown on standard medium (H_2_O) and medium supplemented with 30% D_2_O.

HSP70 proteins are essential for protein folding, processing, and transport, and directly regulate ROS metabolism by interacting with ROS metabolism enzymes [37]. We hypothesized that the HSP70 isoforms produced to compensate for the *hsp70* mutation could impact ROS metabolism. To test this hypothesis, we analyzed superoxide production in seedlings grown on 30% (*v*/*v*) D_2_O using histochemical staining (Figure 6a,b). The content of superoxide correlated well with the observed alleviation of D_2_O-induced growth inhibition, showing a significant increase in superoxide radicals in *hsp70* mutants compared to Col-0. This finding seemingly supports the critical role of ROS metabolism in the D_2_O response, but it could also reflect promoted photosynthetic activity.

## 3. Discussion

Our experiment has inherent limitations originating from the comparison of three contrasting model organisms. Three-hour-incubation at 28 °C represents approximately three doubling times for *E. coli* and about half of that for *S. cerevisiae*. Roots of *Arabidopsis* seedlings grown at 20 °C exhibit average growth rates around 100 µm per hour and the average cell cycle duration is estimated at 18.6 h [38]. We have minimized at least a part of the experimental bias by comparing relative fold changes to respective controls grown under optimal conditions and avoided absolute comparisons that would likely reflect differences in the life style and adaptations. However, the differences between single-cell microbes and multicellular plant model remained. We analyzed whole seedlings and, thus, any tissue-specific effect is lost in our experiment. Further, a part of the limitation would be in a possibility of a contrasting uptake of heavy water. This question was addressed in the analysis of deuterium incorporation into primary metabolites that was comparable (Figure 1) and its speed seemed well in line with the previously published results [12].

The set timeframe of the experiment was shorter than previously reported experiments with heavy water, yet our objective was to assess the early response on the proteome level and three hours is a timepoint that reflects new protein synthesis on the global proteome level [39], and is below the range of half-life for most *E. coli* proteins [40]. Given the rapid incorporation of deuterium atoms into amino acids, this timepoint was also optimal as the deuterium incorporation into proteins was not sufficient to impact algorithms for peptide-based quantitation and none of the evaluated peptides showed a significant presence of deuterium atoms in its molecule that would alter quantitation based on three most intensive isotopologues.

### 3.1. Photosynthesis Could Be One of the Reasons for the Deteriorative Effect of Heavy Water on Arabidopsis

Proteome analysis reveals an accumulation of photosynthesis-related proteins in response to heavy water exposure (Table S5). Such accumulation is commonly associated with damage to the photosystems and impairment of the photosynthetic apparatus, as observed in freezing stress experiments [41]. However, our data do not indicate any significant imbalance in the plant’s reactive oxygen species (ROS) metabolism, which would suggest corresponding damage (Figure 4). Previous studies on algae have shown that overall photosynthesis rates decrease by 20–30% during culture in D_2_O, with the initial effects of heavy water being confined to photosynthetic reactions, while prolonged exposure impairs both respiration and photosynthesis [42]. It is, therefore, more plausible that the observed accumulation of photosynthesis-related proteins results from a reduction in reaction rates due to the interaction with heavy water. This hypothesis is further supported by the notable incorporation of deuterium into the sucrose molecule (Figure 1), the primary carbohydrate transport form produced by photosynthesis. This likely poses a disadvantage to autotrophic organisms, as the photolysis of D_2_O accelerates its integration into plant metabolism, in contrast to bacteria and yeast, which primarily utilize energy from non-deuterated sources in the medium.

### 3.2. Ribosome Remodeling Is a Conserved Response to Heavy Water in Yeast, Bacteria, and Plant

Ribosomal proteins, integral structural components of the ribosome, form a highly dynamic molecular machine that is central to protein synthesis. Contrary to being a static complex or merely a passive participant in translational regulation, ribosomes are highly adaptable, with each ribosome incorporating only one protein from families of multiple paralogues. According to the Kyoto Encyclopedia of Genes and Genomes (KEGG, accessed August 2024), the yeast *Saccharomyces cerevisiae* and *E. coli* genomes encode genes for 197 and 56 ribosomal proteins, respectively. Of these, 103 and 53 ribosomal proteins were quantified in our datasets (Supplementary Tables S3 and S4). This suggests a degree of functional redundancy, with some genes being non-essential under standard conditions. Nonetheless, non-lethal loss-of-function mutations in ribosomal protein genes have been linked to growth defects, as well as reduced cellular, organ, or organismal size [43]. Interestingly, plants exhibit a significantly higher number of ribosomal protein paralogues. The model plant *A. thaliana* harbors over 300 ribosomal protein genes, with at least 225 of these being translated into functional proteins [44], and 142 quantified in our dataset (Supplementary Table S4). Exposure to heavy water resulted in a marked decrease in the abundance of most ribosomal proteins in *E. coli*, aligning with previous reports of growth inhibition under D_2_O treatment [8]. In contrast, the yeast proteome showed an increase in abundance for twenty ribosomal proteins (although only four met the fold-change threshold of 1.4), with only the large subunit ribosomal protein L11 decreasing similarly to what was observed in *E. coli*. The *Arabidopsis* proteome exhibited eight statistically significant changes in ribosomal proteins, notably including a decrease in ribosomal protein L11 (Supplementary Figure S1). Previous studies have documented the induced accumulation of specific ribosomal protein paralogues in response to various stimuli, such as UV light [45], nutrient availability [46], sucrose [47], and cold response [48]. Similar changes were observed in plants with altered cytokinin homeostasis [49,50], as well as in response to hydrogen peroxide [51], salinity, and water deprivation [52]. Therefore, the changes in ribosomal protein abundance observed here are likely indicative of a broader reprogramming of cellular functions in response to heavy water.

### 3.3. Proteomic Evidence of Stress Response Activation in Arabidopsis and Putative Mechanisms Activated in Response to Heavy Water

When *A. thaliana* is exposed to an environment containing pure heavy water, global proteome response is relatively mild. However, identified proteins indicate impact on hormonal regulations. Besides polyamine metabolism, which is closely connected to plant hormone cytokinin [50], the identified DAPs included protein related to auxin regulation DRMH1 (decrease in abundance; Supplementary Table S5). The expression of the corresponding gene is repressed by auxin, indicating that D_2_O stimulates the auxin signaling pathway. Auxin is one of the orchestraters of cell wall loosening, a process that regulates cell growth, but also water uptake by a cell [53]. The likely increase in auxin aligns with the accumulation of EXPA1 that causes loosening and extension of plant cell walls. Auxin may be signaling the plant to loosen its cell walls to allow for growth or structural adjustment under the stress of heavy water. The other identified DAPs present a complex and seemingly contradictory response to heavy water stress. The accumulation of osmotic stress-related proteins, such as the dehydrin ERD10, aquaporin PIP1-2, cruciferin 3, and attenuated cell-to-cell communication suggested by the significant decrease in the callose-degrading enzyme BG-PPAP, aligns with the anticipated physiological adaptations to reduced light water availability. However, this response contrasts with the observed decrease in mitogen-activated protein kinase (MAPK6), which regulates abscisic acid response [54] and downregulation of key abscisic acid signaling components (Supplementary Table S5). This paradox may reflect an adaptive mechanism aimed at increasing sensitivity to this phytohormone, potentially as a compensatory response to its impaired transport or biosynthesis. In parallel, attenuated abscisic acid signaling could also account for the observed root growth inhibition (Figure 5), as it is known to act antagonistically to cytokinin—a phytohormone that regulates root growth [49]. However, the conflicting processes could also indicate an unsuccessful attempt to mitigate the detrimental effects of heavy water, ultimately leading to cellular dysfunction and plant death. Alternatively, these results could highlight the limitations of a global proteomic analysis of the entire seedling. A tissue-targeted analysis might be able to provide more specific answers to these questions in the future. Another limitation of our analysis is that it primarily focused on the abundances of highly abundant proteins, potentially overlooking important post-translational modifications as well as additional receptors and transcription factors that may play critical roles in the biological response to D_2_O. By not capturing these less abundant molecules and regulatory modifications, our interpretation lays the groundwork for future studies, but it remains inherently incomplete.

## 4. Materials and Methods

### 4.1. Cell Cultures

*Saccharomyces cerevisiae* (strain Y2HGold, Takara Bio, Kusatsu, Japan) and *Escherichia coli* (strain BL21) cultures were cultivated in standard yeast peptone dextrose (YPD) and lysogeny broth (LB) media, respectively. Log-phase cells (1 mL per sample) were harvested by centrifugation, subsequently resuspended in either light water or heavy water (99.9 atom % D, Millipore, Burlington, MA, USA), YPD or LB media, and incubated at 28 °C with shaking at 400 RPM for 180 min. Next, cells were collected by centrifugation, and flash-frozen in liquid nitrogen. The experimental setup included six biological replicates.

### 4.2. Plant Material

Seeds of *Arabidopsis thaliana* ecotype Col-0 were surface-sterilized and sown on Petri dishes containing half-strength Murashige and Skoog medium with 1% (*w*/*v*) agar. Seeds were stratified at 4 °C for three days, and cultivated at 21 °C/19 °C day/night temperatures, with a 16 h photoperiod (100 μmol m^–2^ s^–1^ photosynthetic photon flux density) for seven days in a growth chamber (AR36LX, Percival Scientific, Perry, IA, USA). Seedlings were cultivated for seven days on half-strength Murashige and Skoog medium. They were then transferred to fresh medium for a 60 min incubation period. Subsequently, seedlings were divided into two groups: one cultivated in medium prepared in light water and the other in heavy water. Both groups were incubated for 180 min. Seedlings were then rapidly harvested, dried, flash-frozen, and ground in liquid nitrogen. The entire experiment was conducted with six biological replicates, each containing approximately 100 seedlings.

Mutant lines *hsp70-1* and *hsp70-5* were obtained from The Nottingham Arabidopsis Stock Centre (SALK_135531C, SAIL_839_A08C1). Plants were cultivated essentially as described above, with one set of media supplemented with 30% (*v*/*v*) D_2_O, and all plates sealed with parafilm to limit evaporation and deuterium exchange with water vapor in the air. The experiment was performed in two biological replicates, each with ten seedlings per sample.

### 4.3. Protein Extraction and Analysis

Bacterial pellets, yeast pellets, and homogenized plant tissues were extracted by sonication in tert-butyl methyl ether:methanol mixture as described previously [55,56]. In brief, samples were lyophilized, plant tissues homogenized using a Retsch mill, and all samples were extracted by sonication in a tert-butyl methyl ether:methanol mixture (3:1). The resulting protein pellets were washed with 80% (*v*/*v*) acetone in water, dried, and solubilized in 8 M urea, 10 mM dithiothreitol, and 100 mM ammonium bicarbonate. Protein content was estimated using a Bradford reagent (Merck, Darmstadt, Germany), and portions of sample corresponding to 100 µg were alkylated and digested with trypsin. The resulting peptides were desalted using VersaPlate C18 (Agilent, Santa Clara, CA, USA). Portions of samples corresponding to 5 μg protein were analyzed by nanoflow reverse-phase liquid chromatography–mass spectrometry using a 15 cm C18 Zorbax column (Agilent), a Dionex Ultimate 3000 RSLC nano-UPLC system, and the Orbitrap Fusion Lumos Tribrid Mass Spectrometer as described previously [57]. The measured spectra were recalibrated and searched against the *S. cerevisiae* (*Saccharomyces cerevisiae* R64-1-1), *E*. *coli* (UP000000625 83333), and *A. thaliana* (Araport 11) databases, and common contaminants databases using Proteome Discoverer 2.5 (Thermo Fisher Scientific, Waltham, MA, USA) employing Sequest HT and MS Amanda 2.0 [58] algorithms. The settings were as follows: enzyme–trypsin, max two missed cleavage sites; MS1 tolerance—5 ppm; MS2 tolerance—0.1 Da, SEQUEST/0.02 Da, MS Amanda; fixed modifications—carbamidomethyl (Cys); dynamic modifications including Met oxidation, Asn/Gln deamidation; and dynamic modifications at the end of the protein—acetylation (N-end); loss of methionine (N-terminus); loss of methionine/acetylation (N-terminus). The resulting peptide hits were filtered for a maximum 1% false discovery rate using the Percolator Node (Proteome Discoverer 2.5). The quantitative analysis centered on (i) proteins identified by two or more unique peptides and (ii) proteins with a single unique peptide but at least ten assigned peptides, aiming for broader proteome coverage.

### 4.4. Metabolome Analysis

Polar fractions of metabolites separated in the initial step of protein extraction were evaporated, dissolved in pyridine, derivatized using N-methyl-N-(trimethylsilyl) trifluoroacetamide, and analyzed using a Q Exactive GC Orbitrap GC–MS/MS mass spectrometer (Thermo Fisher Scientific) coupled to a Trace 1300 Gas chromatograph (Thermo Fisher Scientific) as described previously [59,60]. Metabolites were separated using a TG-5SILMS column (30 m; 0.25 mm; 0.25 μm; Thermo Fisher Scientific) with a temperature gradient (5 min at 70 °C followed by a 9 °C gradient in 1 min to 320 °C and final incubation for 5 min at 320 °C). Helium was used as carrier gas at a constant flow rate of 1.2 mL/min. Metabolites were ionized using the electron ionization mode (electron energy 70 eV, emission current 50 μA, transfer line, and ion source temperature 250 °C). The MS operated in the full scan mode, 60,000 resolution, scan range 50–750 *m*/*z*, automatic maximum allowed injection time with automatic gain control set to 1 × 10^6^. Data were analyzed by Compound Discoverer 3.3 (Thermo Fisher Scientific; peak detection settings—5 ppm; TIC threshold—10,000; S/N threshold—3) and searched against NIST2023, GC-Orbitrap Metabolomics library, and in-house library. Only metabolites that fulfilling identification criteria (score ≥ 80 and ΔRI < 2.5%) were included in the final list of identified compounds. The isotopologue composition was analyzed using FreeStyle 1.6 (Thermo Fisher Scientific).

### 4.5. Histochemical Analyses

The distribution of superoxide radicals was determined as described previously, using ten-day-old seedlings and nitroblue tetrazolium [52]. In brief, seedlings were vacuum-infiltrated with 6 mM nitroblue tetrazolium (Merck) in 10 mM Tris–HCl buffer (pH 7.0) at room temperature for 10 min. Seedlings were then washed in ethanol to remove chlorophyll, and the staining intensity was quantified using ImageJ 1.54j (NIH, https://imagej.net/ij/, accessed 10 August 2024).

### 4.6. Data Analysis and Processing

The reported statistical tests were generated and implemented using the Real Statistics Resource Pack software for MS Excel (Release 6.8; Copyright 2013–2020; Charles Zaiontz; www.real-statistics.com, accessed 10 August 2024), MetaboAnalyst 6.0 [61], SIMCA 14.1 (Sartorius, Göttingen, Germany), Proteome Discoverer 2.5 (Thermo Fisher Scientific), and CompoundDiscoverer 3.0 (Thermo Fishe Scientific). Significant differences refer to *p* < 0.05, adj. *p*-value represents Benjamini and Hochberg procedure at 5% FDR. Microsoft Excel and PowerPoint were used for data visualization, except where otherwise specified in the respective figures.

## 5. Conclusions

The primary objective of this study was to elucidate the molecular mechanisms that respond to heavy water in *E. coli* and *S. cerevisiae*, and to compare these with those in *Arabidopsis thaliana*, which shows an impaired ability to survive in media containing more than 70% D_2_O. Our initial observations reveal distinct patterns of deuterium incorporation into primary metabolites. Specifically, deuterium incorporation in *Arabidopsis* was slower in the five selected amino acids compared to *S. cerevisiae*, though it did not significantly differ (*p* < 0.05) from that observed in bacteria. Notably, *Arabidopsis* accumulated high levels of deuterium-labeled sucrose, a phenomenon not observed in the microbes. This accumulation, along with the observed increase in photosynthesis-related proteins, suggests an enhanced demand for photosynthesis, potentially leading to increased deuterium incorporation into metabolic processes. This may contribute to the impaired survival in heavy water. However, the ability of algae and some mosses to thrive in high D_2_O concentrations [14] suggests that deuterium incorporation via photosynthesis alone is not the sole factor influencing survival. A critical distinction appears to be the absence of a significant impact on ROS metabolism in *Arabidopsis*, which does not seem to align with typical abiotic stress responses. This may be linked to the impaired abscisic acid signaling observed in our data. Additionally, the activation of chaperones in *Arabidopsis* was markedly lower than in bacteria or yeast. Analysis of mutant lines *hsp70-1* and *hsp70-5* confirmed that modulating the chaperone pool can at least partially alleviate D_2_O-induced root inhibition. Collectively, these findings indicate that *Arabidopsis* fails to survive in heavy water due to defective stress sensing and maladaptive responses to the stressor.

Metabolic flexibility is a critical criterion for survival across diverse environmental conditions [62]. However, as biological systems increase in complexity, this flexibility tends to decrease, as more intricate organisms, such as multicellular eukaryotes, require precise homeostasis within individual cells as well as coordinated intercellular communication. High D_2_O concentrations represent an environmental stressor for which organisms lack specific evolutionary adaptations, suggesting their responses may be inherently non-specific. Our data indicate that fundamental biological processes, including protein, amino acids, and nucleotide synthesis, are affected by D_2_O in a similar manner across species. This shared impact suggests that these early effects may represent a generalizable response to stimuli across all organisms. We observed species-specific variations in proteomic response magnitude, with notable differences across organisms. Interestingly, in *Arabidopsis*, despite rapid deuterium incorporation into metabolites (Figure 1), the early proteome response rate did not match the known toxicity, indicating a lag in cellular adaptation. Seemingly counterintuitively, as organismal complexity increased, the rate of response to D_2_O exposure decreased. Differentially abundant proteins comprised 27.5% of the estimated proteome in bacteria, 3.6% in yeast, and less than 2% in *Arabidopsis*. However, the high impact on bacterial proteome is consistent with both the higher metabolic flexibility of bacteria and their comparatively greater resilience to D_2_O. Our study examined only three organisms spanning different levels of biological complexity. Whether the observed trend applies broadly across other taxa and persists under prolonged D_2_O exposure remains a critical avenue for future research.

## Data Availability

The original contributions presented in the study are included in the article/Supplementary Materials, further inquiries can be directed to the corresponding author.

## Supplementary Materials

The following supporting information can be downloaded at: https://www.mdpi.com/article/10.3390/plants13223121/s1, Figure S1: Differentially abundant ribosomal proteins; Table S1: Deuterium incorporation into primary metabolites following three hours of D_2_O exposure; Table S2: Identified metabolites and estimated relative abundances in three model organisms; Table S3: Quantified proteins identified in *E. coli*; Table S4: Quantified proteins identified in *S. cerevisiae*; Table S5: Quantified proteins identified in *A. thaliana*; Table S6: Results of OPLS/VIP analysis; Table S7: Reactive oxygen species metabolism.
