# Supplementary figures and images for: Divergent Molecular Responses to Heavy Water in Arabidopsis thaliana Compared to Bacteria and Yeast
Source: Plants (Basel). 2024 Nov 6;13(22):3121. doi: 10.3390/plants13223121 (PMC11597629; doi:10.3390/plants13223121)

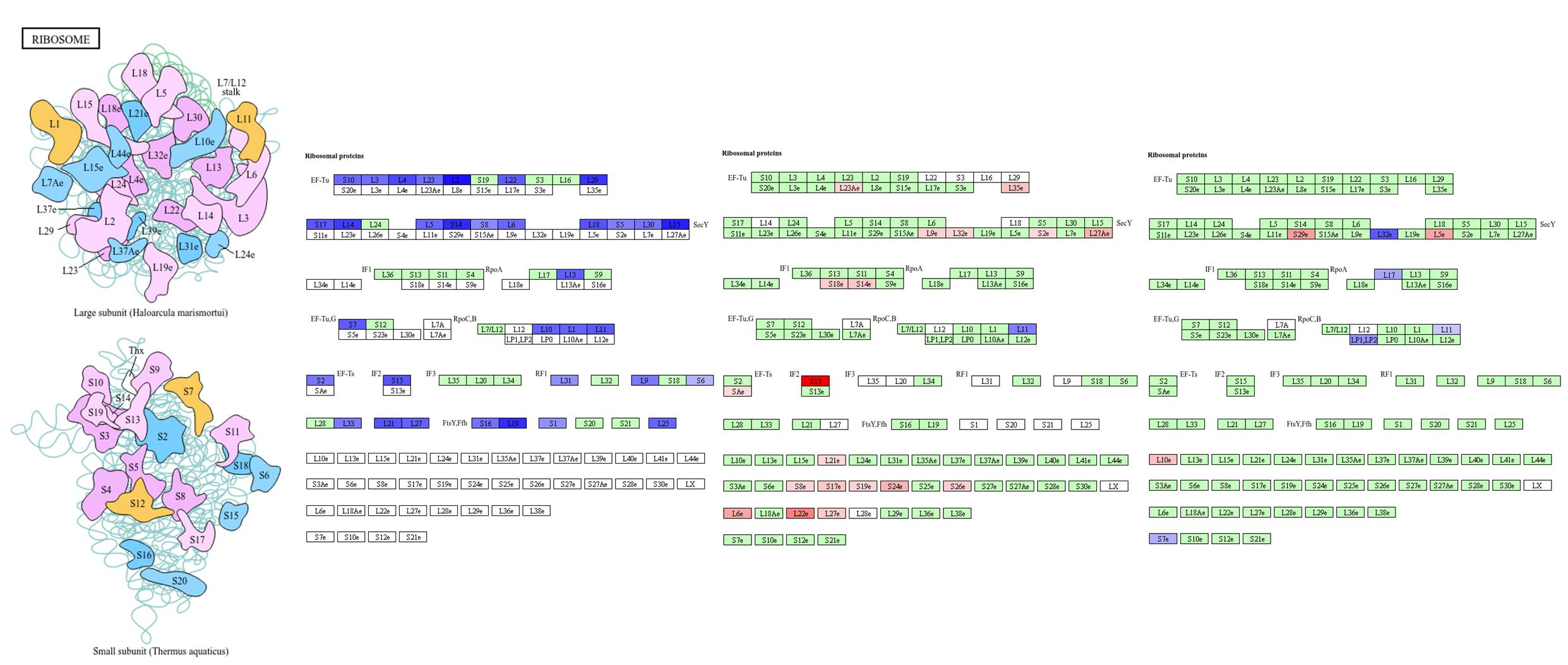

Supplement: Supplementary file 1 [file plants-13-03121-s001.zip › FigureS1.jpg]
